# Investigating the molecular mechanism of Yangxin decoction in treating major depressive disorder using network pharmacology and molecular docking technology approaches

**DOI:** 10.1097/MD.0000000000045050

**Published:** 2025-10-17

**Authors:** Penggang Li, Chai Nien Foo, Yang Mooi Lim

**Affiliations:** a Centre for Cancer Research, M. Kandiah Faculty of Medicine and Health Sciences, Universiti Tunku Abdul Rahman, Kajang, Selangor, Malaysia; b Department of Population Medicine, M. Kandiah Faculty of Medicine and Health Sciences, Universiti Tunku Abdul Rahman, Kajang, Selangor, Malaysia; c Department of Pre-clinical Science, M. Kandiah Faculty of Medicine and Health Sciences, Universiti Tunku Abdul Rahman, Kajang, Selangor, Malaysia.

**Keywords:** major depressive disorder, molecular docking, molecular mechanism, network pharmacology, pharmacological effects, Yangxin decoction

## Abstract

Yangxin decoction has been used to treat major depressive disorder (MDD). This study aims to identify the active components and potential mechanisms of Yangxin decoction in treating MDD using network pharmacology and molecular docking technology. The active components and targets of Yangxin decoction were screened, and MDD-related targets were predicted. Networks of “herbal medicine-active components-potential targets” and protein–protein interaction were constructed. Core components and core targets were identified through network topology analysis. Gene ontology functional and Kyoto Encyclopedia of Genes and Genomes pathway enrichment analyses were performed on candidate genes. Molecular docking was conducted using AutoDock software (Olson Laboratory of the Scripps Research Institute, San Diego) to explore the interactions between core targets and active components, and the results were visualized using PyMOL (DeLano Scientific LLC, South San Francisco). A total of 433 active components and 392 targets of Yangxin decoction were identified, along with 11,796 MDD-related targets. There were 680 overlapping targets between Yangxin decoction and MDD, associated with 104 active components. Core targets identified through network topology analysis and molecular docking included serine/threonine kinase 1 (AKT1), tumor necrosis factor, interleukin-6, tumor protein P53, and proto-oncogene tyrosine-protein kinase Src. Gene ontology enrichment analysis revealed 1606 biological processes, 191 cellular components, and 373 molecular functions. Kyoto Encyclopedia of Genes and Genomes pathway analysis identified 212 signaling pathways, with significant enrichment in caffeine metabolism, bladder cancer, advanced glycation end products-receptor for advanced glycation end products signaling pathway in diabetic complications, and vascular endothelial growth factor signaling pathway. Molecular docking results showed strong binding energy between core active components and core targets. Yangxin decoction exhibits multi-component, multi-pathway, and multi-target therapeutic characteristics. It primarily regulates targets such as AKT1, tumor necrosis factor, interleukin-6, tumor protein P53, and proto-oncogene tyrosine-protein kinase Src through advanced glycation end products-receptor for advanced glycation end products, vascular endothelial growth factor, and ErbB signaling pathways, exerting anti-inflammatory, immune-regulating, and oxidative stress-inhibiting effects to alleviate MDD.

## 1. Introduction

Major depressive disorder (MDD) is a central nervous system disorder characterized by prolonged and persistent low mood and delayed responses, posing a severe threat to human health. A significant number of patients suffer disability or death due to suicidal behaviors,^[1]^ making it a heavy burden both in China and globally. The pathogenesis of MDD is complex and not fully understood, with factors such as psychological, physiological, and social influences contributing to its onset. Widely accepted hypotheses regarding MDD mechanisms include the monoamine neurotransmitter hypothesis, hypothalamic–pituitary–adrenal axis hyperactivity, glutamate receptor hypothesis, neurotrophic hypothesis, neuroplasticity hypothesis, cytokine hypothesis, and gut microbiota hypothesis.^[2]^ In traditional Chinese medicine (TCM), MDD falls under the category of “Yu Zheng” (depression syndrome). Ancient medical texts, such as the *Yellow Emperor’s Classic of Internal Medicine*, provide early insights into the emotional and etiological aspects of MDD, with the “Five Depressions Theory” being the earliest conceptual framework. The primary causes of Yu Zheng are emotional imbalances, particularly sadness, excessive worry, and anger.^[3]^

Yangxin decoction, 1st recorded in Renzhai Zhizhi Fang (Volume 11),^[4]^ is traditionally used to treat palpitations and anxiety due to heart blood deficiency and heart spirit instability. Modern practitioners have expanded its application to other conditions with similar pathogenesis, such as insomnia and angina.^[4]^ Both clinical and basic research have demonstrated that Yangxin decoction effectively alleviates depressive symptoms, improves TCM syndromes, and enhances neurotransmitter transmission in the brain.^[5]^

This study employs network pharmacology and molecular docking to explore the active components, targets, and pathways of Yangxin decoction in treating MDD, providing new insights and theoretical foundations for its clinical application.

## 2. Materials and methods

### 2.1. Prediction of active components and targets of Yangxin decoction

The chemical components of Radix Astragali, Poria, Rhizoma Pinelliae, Radix Angelicae Sinensis, Rhizoma Chuanxiong, Semen Biotae, Semen Ziziphi Spinosae, Fructus Schisandrae, Radix Ginseng, Radix Glycyrrhizae, Poria cum Radix Pini, Radix Polygalae and Cortex Cinnamomi were retrieved from the TCMSP database (https://tcmsp-e.com/).^[6]^ Active components were screened based on oral bioavailability ≥ 30% and drug-likeness ≥ 0.18, and their corresponding targets were obtained. The HERB database (http://herb.ac.cn/)^[7]^ was used to supplement the chemical components of Poria cum Radix Pini, Radix Polygalae, and Cortex Cinnamomi. After deduplication, the targets were standardized using the UniProt database (https://www.uniprot.org/)^[8]^under the conditions of *Homo sapiens* and “reviewed,” resulting in standardized gene symbols.

### 2.2. Collection of MDD-related targets

Using “Depression” as the keyword, targets related to depression were retrieved from GeneCards (https://www.genecards.org/),^[9]^ OMIM (https://omim.org/),^[10]^ TCMSP (https://tcmsp-e.com/),^[6]^ and TTD (http://db.idrblab.net/ttd/).^[11]^ After merging and deduplication, the MDD-related disease targets were obtained.

### 2.3. Identification of potential targets for Yangxin decoction in treating MDD

The targets of Yangxin decoction and MDD-related targets were input into the bioinformatics platform (http://www.bioinformatics.com.cn/), and a Venn diagram was used to identify overlapping targets, which were considered potential targets for Yangxin decoction in treating MDD.

### 2.4. Construction of the herb-active component-potential target network

The potential targets, corresponding active components, and herbs were imported into Cytoscape 3.10.0 to construct the network. The analyze Network plugin was used to calculate topological parameters and identify key active components and targets.

### 2.5. Protein–protein interaction (PPI) network analysis and core target screening

The overlapping targets were input into the STRING 12.0 database (https://cn.string-db.org/),^[12]^ with the species set to *H sapiens* and a confidence threshold of 40%. The PPI network was constructed, and the cytoNCA plugin^[13]^was used for topological analysis. Core targets were identified based on median values of betweenness centrality (BC), closeness centrality (CC), and degree centrality (DC). The results were visualized using Cytoscape 3.10.0.

### 2.6. Gene ontology (GO) and Kyoto Encyclopedia of Genes and Genomes (KEGG) pathway enrichment analysis

The overlapping targets were input into the Metascape database (https://metascape.org/gp/index.html#/main/step1.),^[14]^ with the species set to *H sapiens* and the analysis mode set to “Custom Analyze.” GO and KEGG pathway enrichment analyses were performed with a significance threshold of *P* ≤ .01. The results were visualized using the Weisen Bioinformatics Platform and Cytoscape 3.10.0.

### 2.7. Molecular docking

The top 5 active components with degree values > 20 and the top 5 core proteins from the PPI network were selected for molecular docking. The 3D structures of target proteins were obtained from the PDB database (http://www.rcsb.org/), UniProt (https://www.uniprot.org/), and TCMSP (https://tcmsp-e.com/). The 2D structures of active components were converted and energy-optimized using Chem3D (Revvity, Inc. [Revvity Signals], Waltham) and PyMOL (DeLano Scientific LLC, South San Francisco). AutoDockTools 1.5.7 (Olson Laboratory of the Scripps Research Institute, San Diego) was used for molecular docking, and the results with the lowest binding energy were selected as the optimal conformations.^[15]^

## 3. Results

### 3.1. Active components and targets of Yangxin decoction

Based on the screening criteria, 17 active components from Radix Astragali, 6 from Poria, 12 from Rhizoma Pinelliae, 3 from Radix Angelicae Sinensis, 6 from Rhizoma Chuanxiong, 5 from Semen Biotae, 7 from Semen Ziziphi Spinosae, 8 from Fructus Schisandrae, 17 from Radix Ginseng, 88 from Radix Glycyrrhizae, 33 from Poria cum Radix Pini, 57 from Radix Polygalae, and 174 from Cortex Cinnamomi were identified (Tables 1 and 2). After standardization and deduplication using the UniProt database, a total of 1073 target genes were obtained.

**Table 1:** Active ingredients of Yangxin decoction and the number of their targets.

Chinese medicine	Component/piece	Target/piece
Huang Qi	17	462
Fu Ling	6	21
Ban Xia	12	159
Dang Gui	3	71
Chuan Xiong	6	42
Bai Zi Ren	5	45
Suan Zao Ren	7	46
Wu Wei Zi	8	30
Ren Shen	17	256
Gan Cao	88	1769
Fu Shen	33	1620
Yuan Zhi	57	2537
Rou Gui	174	4738

**Table 2:** Bilingual list of traditional Chinese medicine names.

Pinyin	Latin name
Huangqi	Radix Astragali
Fuling	Poria
Banxia	Rhizoma Pinelliae
Danggui	Radix Angelicae Sinensis
Chuanxiong	Rhizoma Chuanxiong
Baiziren	Semen Biotae
Suanzaoren	Semen Ziziphi Spinosae
Wuweizi	Fructus Schisandrae
Renshen	Radix Ginseng
Gancao	Radix Glycyrrhizae
Fushen	Poria cum Radix Pini
Yuanzhi	Radix Polygalae
Rougui	Cortex Cinnamomi

### 3.2. Active ingredients and targets of Yangxin decoction in MDD intervention

**Figure 1.**
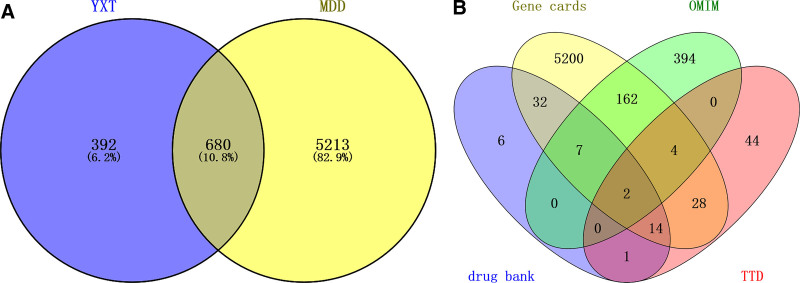
Mapping of key targets of Yangxin decoction and major depressive disorder. Overlapped genes between targets of active components in Yangxin decoction and major depressive disorder related genes from the GeneCards, DrugBank, OMIM, TTD databases. (A) Major depressive disorder related genes; (B) overlapped genes. MDD = major depressive disorder, OMIM = online Mendelian inheritance in man database, TTD = therapeutic target database, YXT = YangxinTang.

By searching the GeneCards, OMIM, TCMSP, and TTD databases and removing duplicates, a total of 5893 MDD-related targets were identified. As shown in Figure 1, 680 overlapping genes between the drug and the disease were obtained using the online analysis platform Venny 2.1.0 (BioinfoGP [CNB-CSIC], Madrid, Spain), representing the potential targets of Yangxin decoction in MDD intervention.

**Figure 2.**
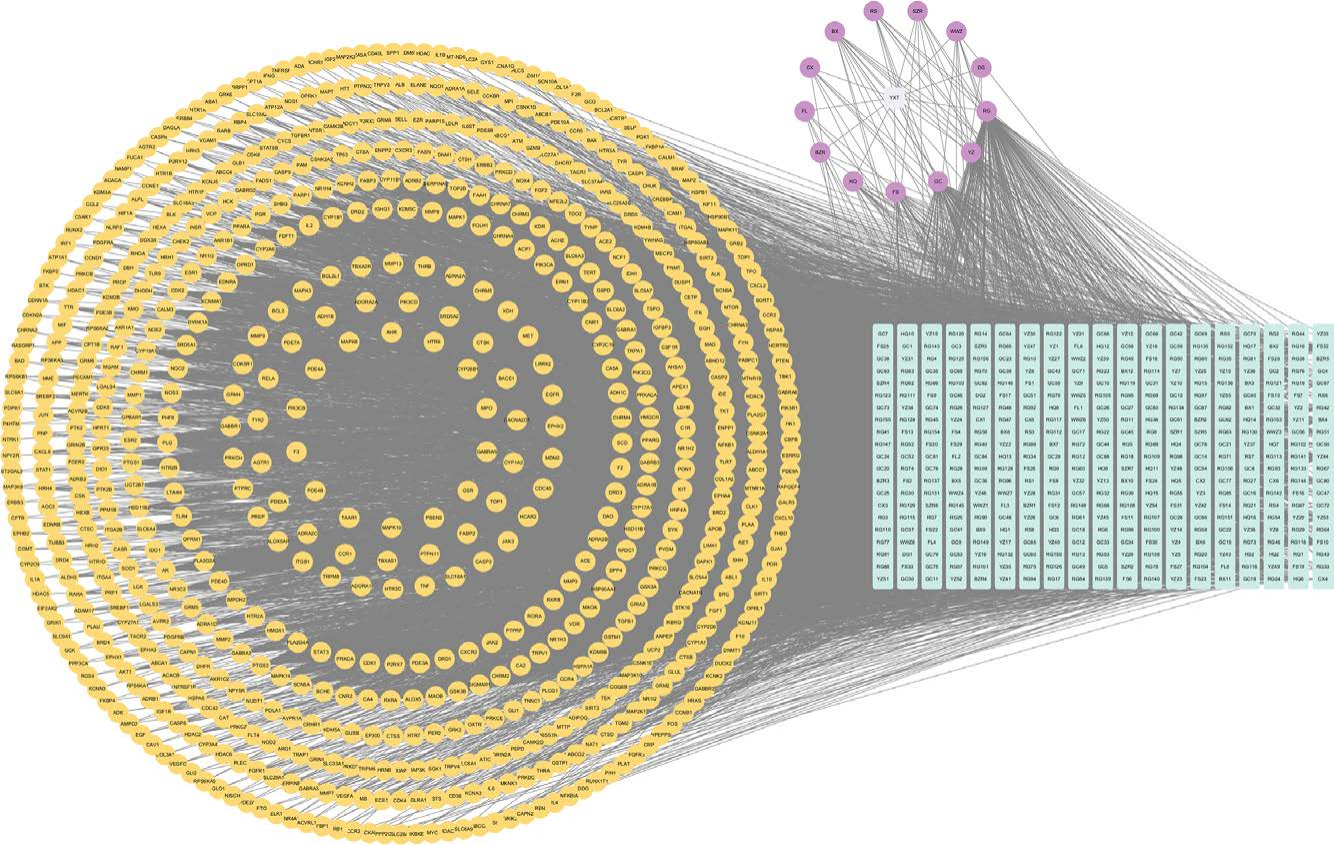
Yangxin decoction “herb-active compound-potential target network.” Network diagram of active compounds and targets of Yangxin decoction in the treatment of major depressive disorder. Purple circles represent the traditional Chinese medicines contained in Yangxin decoction, cyan rectangles represent the active compounds, and orange circles represent the targets related to major depressive disorder (MDD). The lines indicate the relationships between the compounds and their corresponding targets.

A herb-active compound-potential target network was constructed using Cytoscape 3.10.0 (Fig. 2). The network consists of 396 nodes and 4368 edges, including 693 potential targets, 400 active ingredients, and 13 herbal medicines. The top 10 ingredients ranked by degree value in the network are listed in Table 3.

**Table 3:**
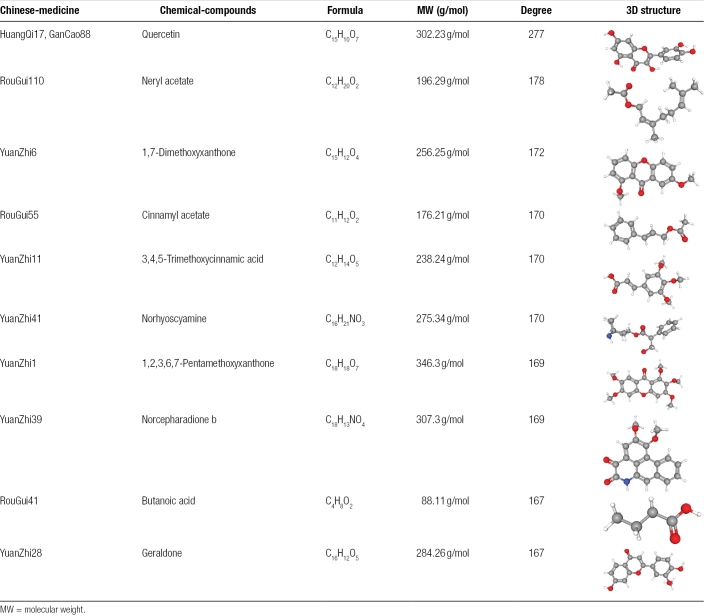
Characteristics of the active ingredients.

### 3.3. PPI network construction and core target screening

**Figure 3.**
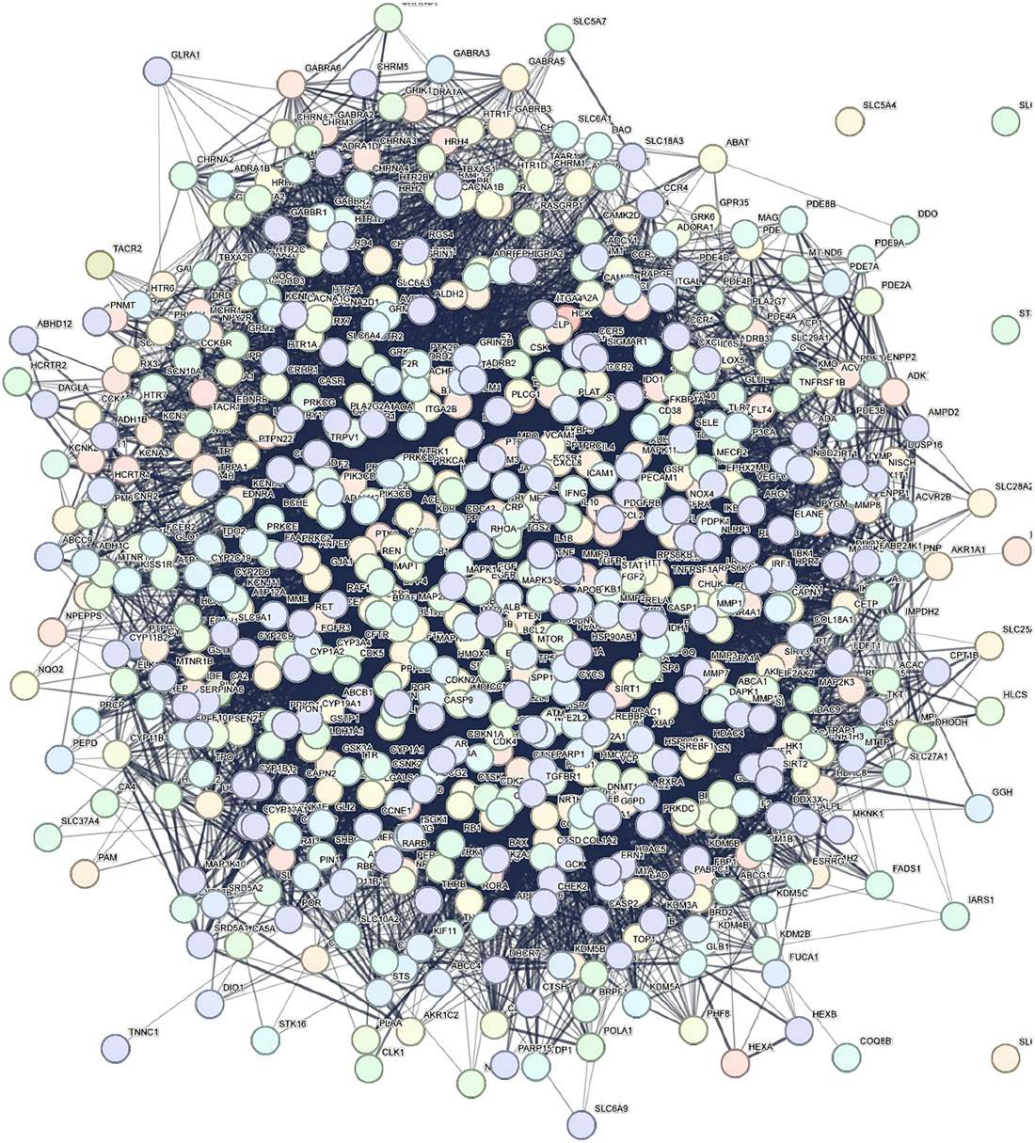
PPI network of major depressive disorder-related targets intervened by Yangxin decoction. PPI = protein–protein interaction.

**Figure 4.**
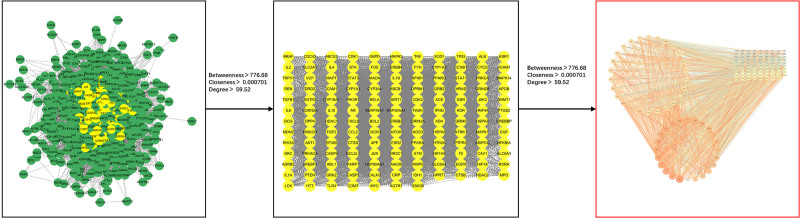
Diagram of core target screening for Yangxin decoction in major depressive disorder intervention.

Using the STRING 12.0 database, a PPI network was constructed by importing 69 intersecting targets. The network was then visually enhanced using Cytoscape 3.10.0, resulting in Network 1 (Fig. 3). Topological analysis of Network 1 was conducted using cytoNCA, with the median values of topological parameters BC, DC, and CC as screening criteria. Based on thresholds of BC ≥ 17.22, DC ≥ 40, and CC ≥ 0.71, Network 2 was obtained. Further screening of Network 2 identified the core target network with BC ≥ 776.68, DC ≥ 0.000701, and CC ≥ 59.52. The results revealed 10 key targets with high degree values in the network: serine/threonine-protein kinase 1 (AKT1), tumor necrosis factor (TNF), interleukin-6, tumor protein p53 (TP53), Proto-oncogene tyrosine-protein kinase Src (SRC), interleukin-1 beta, albumin, signal transducer and activator of transcription 3, transcription factor Jun, and epidermal growth factor receptor (Table 4). These targets represent the core mechanisms through which Yangxin decoction intervenes in MDD (Fig. 4).

**Table 4:** Characteristics of the top 10 hub gene.

Uniprot ID	Gene symbol	Protein name	Degree
P31749	AKT1	RAC-alpha serine/threonine-protein kinase	341
P01375	TNF	Tumor necrosis factor	320
P05231	IL6	Interleukin-6	316
P04637	TP53	Cellular tumor antigen p53	305
P12931	SRC	Proto-oncogene tyrosine-protein kinase Src	293
P01584	IL1B	Interleukin-1 beta	288
P02768	ALB	Albumin	288
P40763	STAT3	Signal transducer and activator of transcription 3	269
P05412	JUN	Transcription factor Jun	257
P00533	EGFR	Epidermal growth factor receptor	255

### 3.4. GO functional and KEGG pathway enrichment results

**Figure 5.**
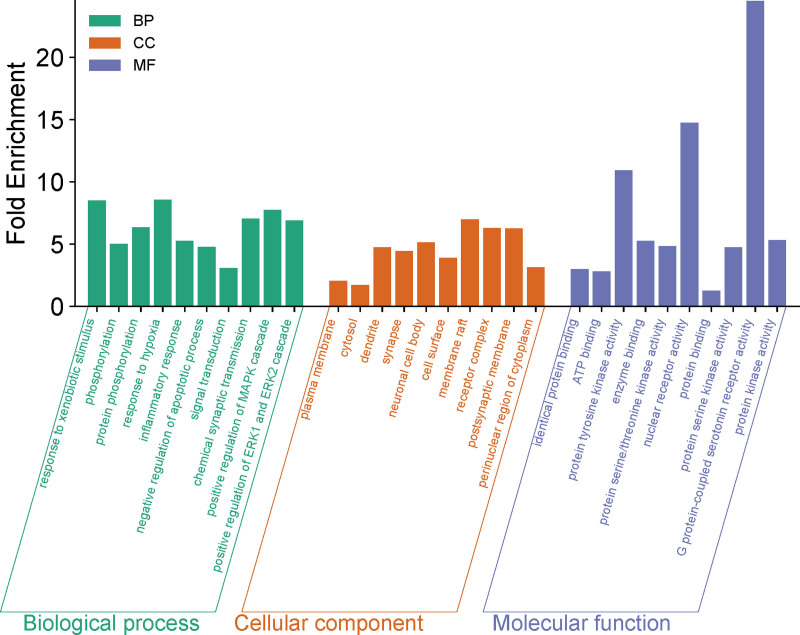
Bar chart of GO enrichment analysis of therapeutic targets for molecular function. The ontology enclosed 3 domains: biological process (green), cellular component (orange), and molecular function (blue). BP = biological process, GO = gene ontology, MF = molecular function.

GO functional enrichment analysis identified 1606 biological processes (BP), 191 cellular components, and 373 molecular functions. The results indicated that the intersecting targets are primarily involved in BP such as response to xenobiotic stimulus, phosphorylation, protein phosphorylation, response to hypoxia, inflammatory response, and negative regulation of apoptotic process, etc. The targets are mainly distributed in cellular components such as the plasma membrane, cytosol, dendrite, synapse, and neuronal cell body, etc. The molecular functions include identical protein binding, ATP binding, protein tyrosine kinase activity, enzyme binding, and protein serine/threonine kinase activity, etc. A bar chart was generated to illustrate the top 10 terms ranked by *P*-value (Fig. 5).

**Figure 6.**
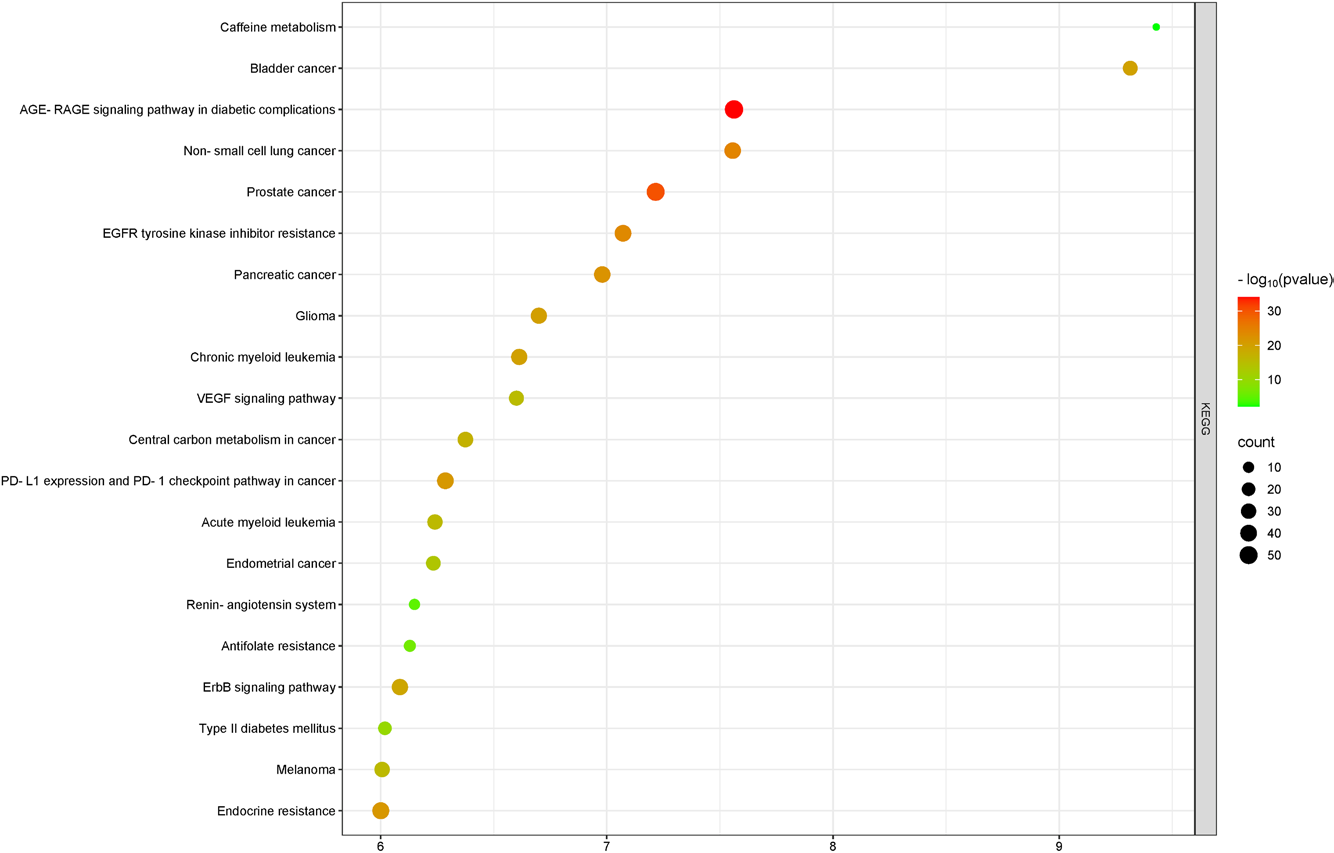
Bubble chart of KEGG enrichment analysis for therapeutic targets. The gene ratio is represented by *X*-axis, the enriched pathways is represented by the *Y*-axis; the gene number is indicated by dots size; the level of *P*-value is represented by dots color. AGE-RAGE = advanced glycation end products-receptor for advanced glycation end products, EGFR = epidermal growth factor receptor, KEGG = Kyoto Encyclopedia of Genes and Genomes, VEGF = vascular endothelial growth factor.

**Figure 7.**
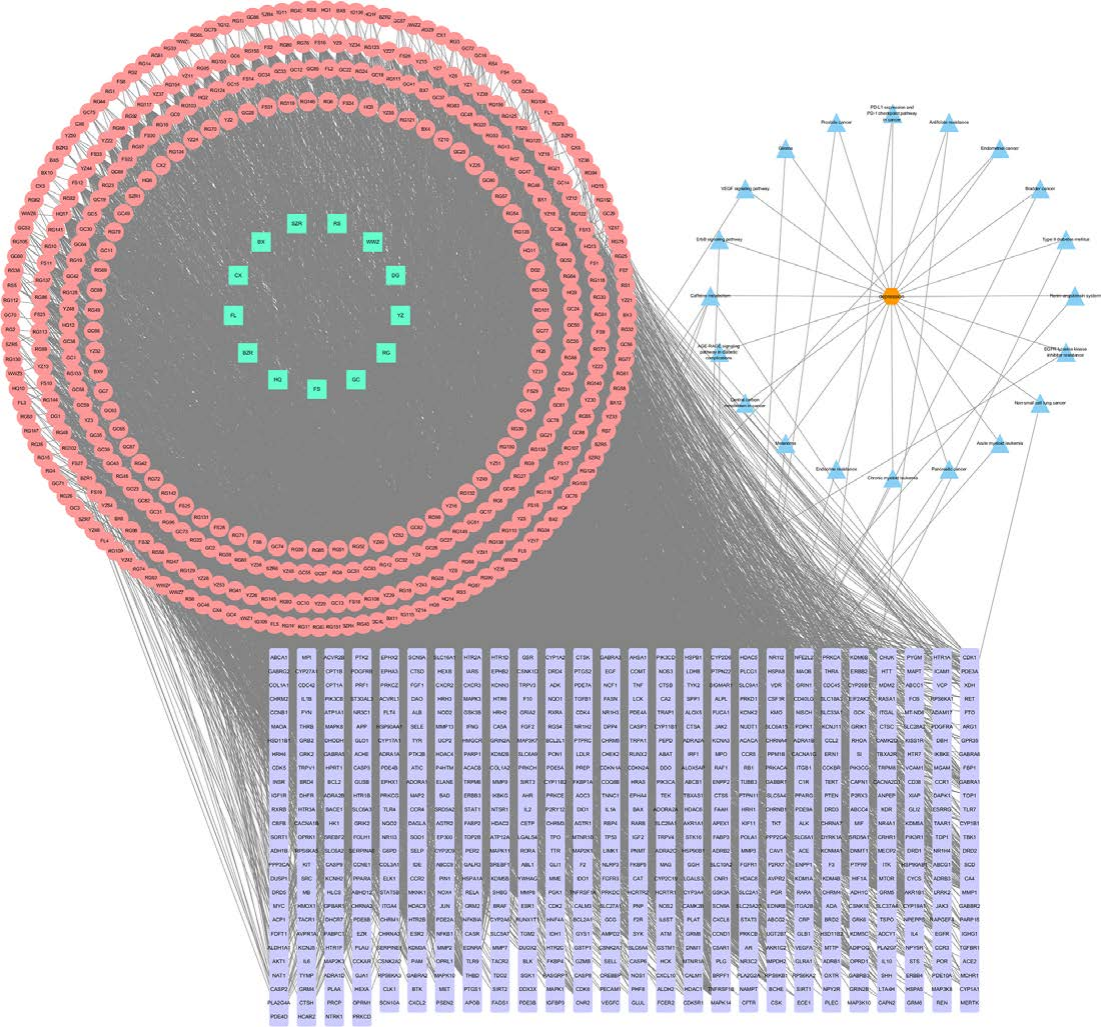
Herb-active compound-disease-intersecting target-pathway network diagram. The cyan rectangles represent the herbal medicines contained in Yangxin decoction, the pink circles denote the active compounds, and the lavender rectangles indicate the targets associated with major depressive disorder (MDD). The blue triangles symbolize the relevant pathways, while the connecting lines illustrate the interactions among the herbs, active compounds, targets, and pathways.

KEGG pathway enrichment analysis identified 212 pathways. The results showed that the intersecting targets were primarily enriched in pathways related to caffeine metabolism, bladder cancer, the role of the advanced glycation end products-receptor for advanced glycation end products (AGE-RAGE) signaling pathway in diabetic complications, non-small cell lung cancer, prostate cancer, epidermal growth factor receptor tyrosine kinase inhibitor resistance, pancreatic cancer, vascular endothelial growth factor (VEGF) signaling pathway, glioma, chronic myeloid leukemia, and acute myeloid leukemia. A bubble chart was created to display the top 20 pathways ranked by *P*-value (Fig. 6). Furthermore, the 20 core pathways associated with Yangxin decoction’s intervention in MDD, along with 680 intersecting targets, were used to construct a “Herb-active compound-disease-intersecting target-pathway” network using Cytoscape 3.10.0 (Fig. 7). The network consists of 1113 nodes and 8514 edges, where cyan, lavender, and blue represent herbs, intersecting targets, and core pathways, respectively.

### 3.5. Molecular docking results

**Figure 8.**
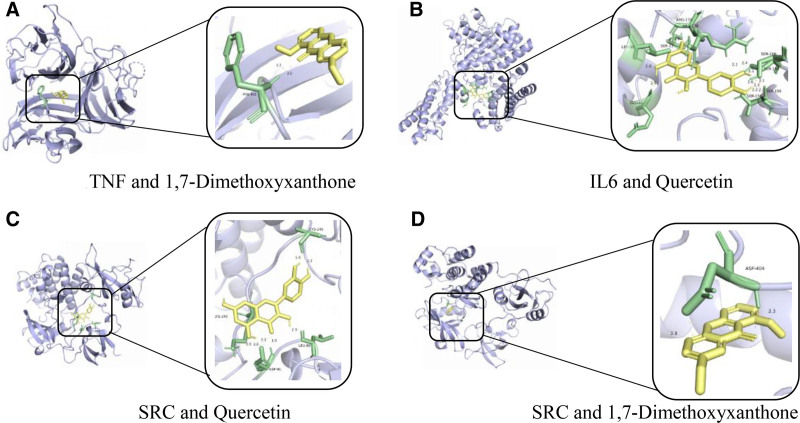
Molecular docking conformations of core target proteins and active compounds in Yangxin decoction for major depressive disorder intervention. Molecular docking of representative compound-target pairs with the strongest affinities. (A) Binding poses of 1,7-dimethoxyxanthone complexed with TNF, affinity = −7.92 kcal/mol; (B) binding poses of quercetin complexed with IL6, affinity = −7.82 kcal/mol; (C) binding poses of quercetin complexed with SRC, affinity = −6.98 kcal/mol; and (D) binding poses of 1,7-dimethoxyxanthone complexed with SRC, affinity = −7.01 kcal/mol. TNF = tumor necrosis factor.

Key core target proteins (AKT1, TNF, IL-6, TP53, and SRC) were docked with AutoDockVina1.1.2 to validate the binding interactions between active compounds and potential targets and enhance the reliability of the target network. Molecular docking analysis was performed on the top 5 core targets (AKT1, TNF, IL-6, TP53, and SRC) and 5 active compounds (Quercetin, Neryl acetate, 1,7-Dimethoxyxanthone, Cinnamyl acetate, and 3,4,5-Trimethoxycinnamic acid) selected based on their DC values, as shown in Tables 3 and 4. The ΔGbind values were predominantly < −5 kcal/mol, indicating strong binding affinities. The results showed that the binding energies of all ligand-receptor interactions were < 0 kcal/mol, indicating good binding affinity between the receptor proteins and ligand molecules (Table 5). The 4 strongest binding interactions were observed between TNF and 1,7-Dimethoxyxanthone, IL-6 and Quercetin, SRC and Quercetin, and SRC and 1,7-Dimethoxyxanthone. These docking results were further visualized using PyMOL (Fig. 8).

**Table 5:**
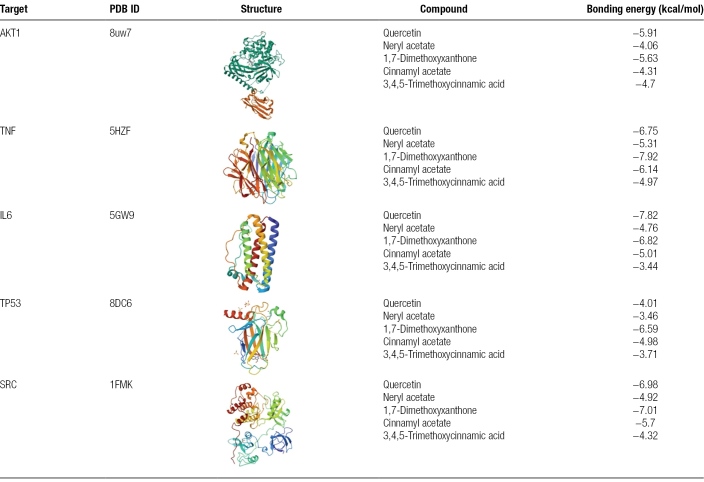
Average binding energy of ligand-receptor molecular Docking (kcal/mol).

## 4. Discussion

MDD, as a chronic disease, not only impairs an individual’s ability to cope with daily life but also coexists with other chronic illnesses, such as cardiovascular diseases and diabetes. This exacerbates damage to normal physiological functions, reduces quality of life, affects prognosis, and increases the risk of mortality.^[16]^ Western medicine treatments for MDD face challenges such as adverse drug reactions, withdrawal effects, and relapse.^[17]^ TCM has a unique therapeutic effect on MDD, not only alleviating clinical symptoms but also improving patients’ tolerance and compliance. TCM formulas have the distinct advantage of acting on multiple layers, targets, and pathways. When used alone for MDD treatment, they can enhance patients’ quality of life without adverse effects, offering a clear advantage over Western medicine.^[18]^

Yangxin decoction is a well-recognized classical formula known for its effectiveness in treating heart-blood deficiency and restlessness of the mind, manifesting as palpitations and anxiety. Modern physicians have expanded its application beyond palpitations and anxiety to other conditions rooted in heart-blood and heart-qi deficiency, such as insomnia and MDD.^[19]^ In this formula, Radix Astragali serves as the principal herb to replenish qi and generate blood, while Rhizoma Chuanxiong and Radix Angelicae Sinensis promote blood circulation. Combined with Radix Astragali, these herbs achieve a balance between tonification and circulation without causing stagnation. Radix Ginseng strongly replenishes vital energy and enhances qi-tonifying effects in conjunction with the principal herb. Poria strengthens the spleen, promoting the generation of qi and blood. Poria cum Radix Pini, Semen Biotae, Fructus Schisandrae, Radix Polygalae, and Semen Ziziphi Spinosae nourish the heart and calm the mind. Cortex Cinnamomi aids in yang promotion and vessel unblocking, while Fructus Schisandrae work together to nourish heart yang and supplement heart yin, achieving yin-yang balance. Radix Glycyrrhizae harmonizes the formula and fortifies the middle burner. Together, these herbs exert a synergistic effect in tonifying qi and blood while calming the heart and mind.^[19]^ Clinical studies have shown that Yangxin decoction significantly improves depressive symptoms in patients. Feng Lu et al^[20]^ reported that modified Yangxin decoction combined with psychotherapy can significantly enhance clinical efficacy in the treatment of depression, alleviate depressive symptoms and TCM syndromes, regulate neurotransmitter levels, and improve patients’ quality of life. Lin Chao^[21]^ study demonstrated that modified Yangxin decoction combined with escitalopram oxalate tablets can effectively improve the psychological status of patients with depression, showing better efficacy than monotherapy with Western medicine. Zhang Qingqing et al^[22]^ further confirmed that this combination therapy not only significantly improves clinical outcomes and psychological well-being, but also enhances quality of life and reduces the incidence of adverse reactions. preclinical studies have shown that Yangxin decoction alleviates depression-like behaviors in CUMS-induced rats, potentially by inhibiting hippocampal neuronal apoptosis and inflammatory responses via the TLR4/NLRP3 signaling pathway.^[23]^

The herb-active compound-potential target network analysis indicated that the active compounds Quercetin, Neryl acetate, 1,7-Dimethoxyxanthone, Cinnamyl acetate, and 3,4,5-Trimethoxycinnamic acid had high degree values and strong associations with core targets such as AKT1, TNF, IL-6, TP53, and SRC. Thus, these 5 active compounds are likely the material basis for Yangxin decoction’s antidepressant effects. Studies have shown that Quercetin alleviates MDD induced by chronic unpredictable mild stress in mice by reducing IL-1β and TNF-α expression in the hippocampus, increasing 5-hydroxytryptamine and brain-derived neurotrophic factor expression, and inhibiting neuroinflammation and apoptosis.^[24]^ Prolonged immobility time in the forced swimming and tail suspension tests indicates extended periods of despair and hopelessness, reflecting weakened willpower, increased negative emotions, and a lack of spontaneous and exploratory behavior in mice. After Quercetin intervention, mice showed a significant increase in body weight and a marked reduction in immobility time, with the 20 mg/(kg·d) dose group showing the most notable improvement. These findings suggest that Quercetin possesses certain antidepressant effects, which are closely related to the dosage. Neryl acetate possesses anti-inflammatory and antioxidant effects and is widely used in the treatment of neurological inflammation and anxiety.^[25]^ Quantitative structure-activity relationship modeling suggests that Neryl acetate may act as an effective antidepressant by binding to dopamine receptor D2, serotonin transporter, and serotonin receptor 1A.^[26]^ It also regulates monoamine oxidase A, preventing serotonin degradation into 5-hydroxyindoleacetic acid, thereby increasing serotonin concentration in the synaptic cleft and alleviating anxiety.^[26]^ 1,7-Dimethoxyxanthone, a Xanthone compound found in *Polygala* species, has potential antidepressant properties.^[27]^ Cinnamyl acetate was identified as one of the most significant active components in Jiaotai Pill, suggesting its role as a primary antidepressant component.^[28]^ 3,4,5-Trimethoxycinnamic acid has antidepressant, anxiolytic, and sedative effects.^[29]^ Research has demonstrated that it induces ΔFosB expression in the nucleus accumbens and increases SC1 expression, improving stress-induced anxiety and MDD-like behaviors.^[30]^

Based on our findings, we hypothesize that the antidepressant effects of Yangxin decoction may be mediated through key molecular targets such as AKT1, TNF, IL-6, TP53, and SRC. Studies suggest that AKT1 polymorphism is associated with MDD severity, anxiety symptoms, work impairment, and suicidal tendencies in patients with MDD.^[31]^ TNF is a key immune regulator.^[32]^ IL-6 plays a biological role in inflammation and immune diseases through multiple pathways.^[33]^ A genome-wide expression study in patients with inflammation-related MDD, particularly comorbid with obesity, identified the TP53 signaling pathway as a distinguishing feature.^[34]^ Serotonin activation of β-arrestin2, Src, and Akt receptor signaling complexes is essential for physiological responses, including head-twitch behaviors. Blocking any single component prevents full expression of these behaviors, indicating the functional relevance of this complex. These insights could significantly impact the targeting of serotonin receptor 2A (5-HT2AR) activation for antidepressant drug development.^[35]^ AKT1 is closely related to apoptosis,^[36]^ and excessive production of inflammatory cytokines like TNF and IL-6 under inflammatory conditions can induce apoptosis.^[37]^ Activation of the AKT pathway can inhibit apoptosis of functional cells, reduce inflammation and oxidative stress, and improve MDD symptoms.^[38]^

KEGG pathway enrichment analysis suggests that the antidepressant effects of Yangxin decoction may involve modulation of the AGE-RAGE, VEGF, and ErbB signaling pathways, as these pathways were significantly enriched among MDD-related genes targeted by the decoction. The RAGE is a pattern recognition receptor involved in pathological processes, including vascular remodeling in the lungs, kidneys, and brain.^[39]^ Recent findings indicate that RAGE binds oxytocin and facilitates its transport into the brain via neurovascular endothelial cells, implicating RAGE signaling in maternal MDD.^[40]^ Previous studies have also demonstrated that natural compounds such as caffeic acid and melatonin alleviate depressive behaviors in rodent models via the AGE-RAGE pathway.^[41]^ Inflammation plays a crucial role in MDD pathogenesis, and the AGE-RAGE pathway regulates inflammation via downstream NF-κB activation and pro-inflammatory cytokine production.^[42]^ VEGF is a vital neurotrophic factor involved in physiological and pathological angiogenesis.^[43]^ It has neurotrophic and neuroprotective properties in both the central and peripheral nervous systems and acts as a mitogen and survival factor for endothelial cells and neurons, regulating synaptic transmission.^[44]^ Studies suggest that VEGF plays a significant role in hippocampal neurogenesis and MDD pathophysiology. Inhibition of VEGF receptor 2 impairs hippocampal-dependent synaptic plasticity and emotional memory consolidation.^[45]^ The ErbB signaling pathway is associated with MDD through neuromodulation and downstream AKT and ERK signaling regulation.^[46]^ Activation of RAGE leads to downstream signaling cascades involving NF-κB, which subsequently upregulates pro-inflammatory cytokines such as TNF and IL-6.^[47]^ Moreover, RAGE stimulation has been shown to influence AKT1 activity through oxidative stress and PI3K/AKT pathway modulation, suggesting that AKT1 may serve as a molecular hub connecting oxidative stress, inflammation, and cell survival in depression.^[48]^ VEGF exerts neuroprotective and neurotrophic effects, partly through activation of the PI3K/AKT1 signaling pathway, which supports neuronal survival and synaptic plasticity. Dysregulation of this pathway may lead to impaired neurogenesis and has been associated with the pathophysiology of depression.^[49,50]^ Additionally, VEGF signaling indirectly modulates inflammatory responses, possibly affecting TNF and IL-6 expression levels.^[51]^ By linking these pathways to AKT1, TNF, and IL-6, the findings support the hypothesis that Yangxin decoction may alleviate depressive symptoms through multi-pathway, multi-target regulation of inflammation, neuroprotection, and cell survival. Future experimental validation focusing on these interactions could further substantiate the proposed mechanisms.

Molecular docking results revealed that all ligand-receptor binding energies were below −1.2 kcal/mol, suggesting potential strong binding affinity. Notably, 1,7-Dimethoxyxanthone exhibited the lowest binding energy with TNF (−7.92 kcal/mol), implying that active components of Yangxin decoction may stably interact with key target proteins. These computational findings propose a plausible mechanism for its antidepressant effects, warranting further experimental validation.

## 5. Conclusions

Thus, the integrated network pharmacology and molecular docking analysis provides multiple testable hypotheses for the molecular mechanism of Yangxin decoction (YXD) against MDD, predicting Quercetin, Neryl acetate,1,7-Dimethoxyxanthone, Cinnamyl acetate, and 3,4,5-Trimethoxycinnamic acid as potential active components of YXD. These components may act on key targets (AKT1, TNF, IL-6, TP53, and SRC) and regulate critical BP and pathways. However, it is important to note that these findings are based solely on in silico predictions and lack experimental or clinical validation, which represents a major limitation of this study. Due to the complex multi-component nature of traditional herbal formulations like YXD, isolating individual chemical components and studying their isolated effects remains challenging. While this network pharmacology approach offers a valuable theoretical framework for understanding YXD’s potential therapeutic effects in MDD, further experimental validation is essential to confirm these predictions. To address these limitations, we plan to combine animal behavioral studies and molecular biology experiments in subsequent research to: elucidate the active material basis of YXD, and systematically investigate its mechanism of action in MDD treatment through in vitro and in vivo validation approaches.

## Author contributions

**Conceptualization:** Penggang Li.

**Data curation:** Penggang Li.

**Formal analysis:** Penggang Li.

**Investigation:** Penggang Li.

**Methodology:** Penggang Li.

**Project administration:** Penggang Li.

**Resources:** Penggang Li.

**Software:** Penggang Li.

**Supervision:** Chai Nien Foo, Yang Mooi Lim.

**Validation:** Penggang Li.

**Visualization:** Penggang Li.

**Writing – original draft:** Penggang Li.

**Writing – review & editing:** Penggang Li.
